# Exploring the Global Health Legacy of Dr. Vulimiri Ramalingaswami: A Review of His Life and Contributions

**DOI:** 10.7759/cureus.65644

**Published:** 2024-07-29

**Authors:** Mayank Sharma, Sonali G Choudhari, Abhishek Ingole

**Affiliations:** 1 Department of Community Medicine, Jawaharlal Nehru Medical College, Datta Meghe Institute of Higher Education & Research, Wardha, IND

**Keywords:** historical vignette, medical scientist, kangra valley, goitre, biography

## Abstract

Dr. Vulimiri Ramalingaswami was an Indian biomedical scientist who rose to fame in the 20th century. He became well-known during his lifetime. Possessing a sharp mind, well-developed communication skills, a love of research and teaching, and a strong commitment to public welfare, he set an unwavering path to success in any field he decided to pursue. He broke new ground in administration, public service, research, and medical education. He was endowed with an enigmatic charm that won the respect of everyone he encountered. He made his students and coworkers leaders by setting a good example. As a result, he excelled in representing Indian biomedical research abroad. He was well known for his ability to think clearly, to present ideas and thoughts either orally or in writing with eloquence, and to do so in a straightforward manner.

## Introduction and background

**Figure 1:**
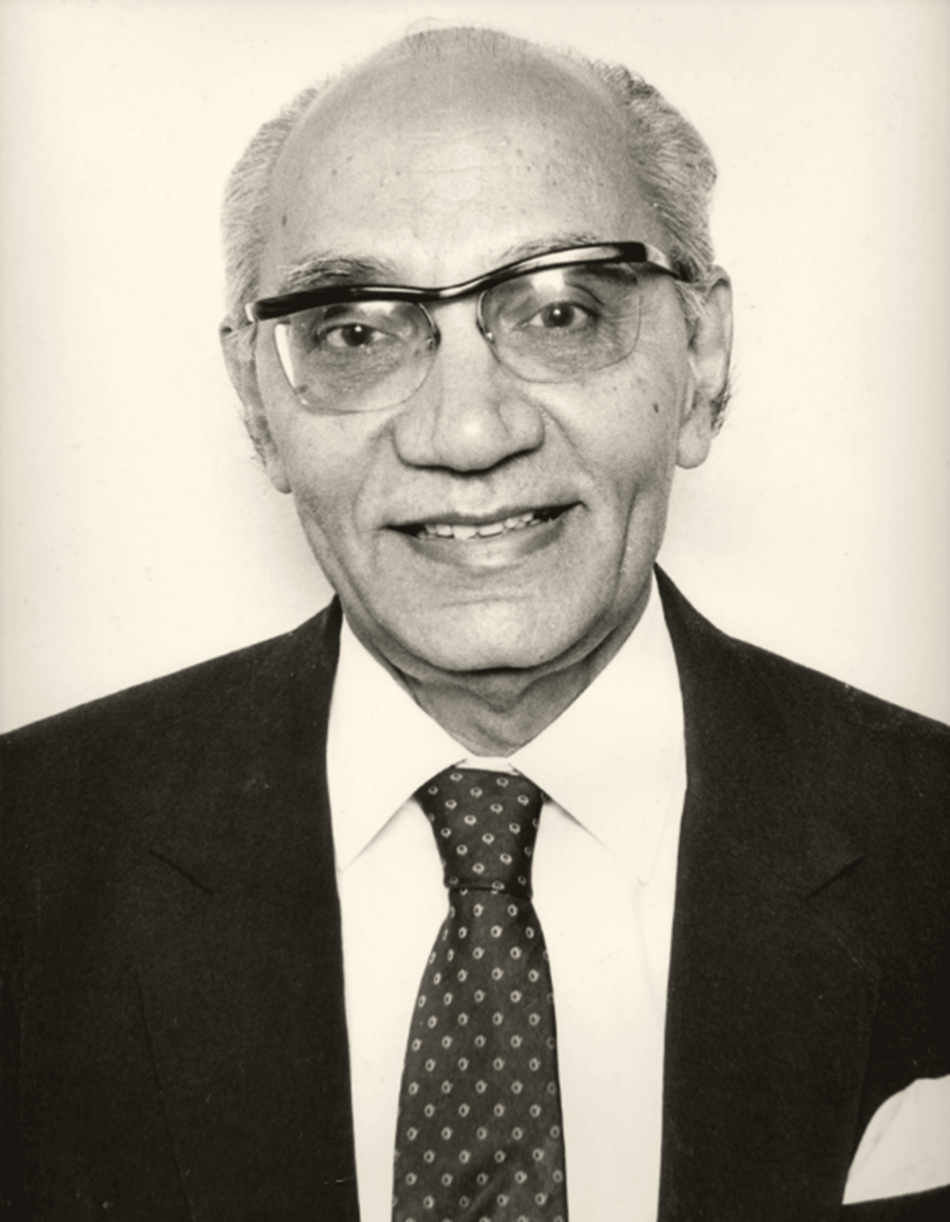
Dr. Vulimiri Ramalingaswami Source [1]

The primary purpose of this article is to highlight the significant contributions of Dr. Vulimiri Ramalingaswami (Figure 1) to global health initiatives, international health, social determinants of health concepts, medical science, public health, and research. He was an Indian medical scientist, pathologist, and medical writer who lived from August 8, 1921, to May 28, 2001. He was elected to the Royal Society of London, the Russian Academy of Medical Sciences, and the National Academy of Sciences for his groundbreaking work in nutrition [1].

In addition, he served as president of the Indian National Science Academy, director general of the Indian Council of Medical Research (ICMR), and director of the All India Institute of Medical Sciences (AIIMS) [2,3]. He was considered an internationally renowned teacher in the field of nutritional deficiency. The Council of Scientific and Industrial Research awarded him the Shanti Swarup Bhatnagar Award in 1967. He was awarded the Government of India's Padma Shri in 1969 and Padma Bhushan in 1971, the first R. D. Birla National Award for Medical Research in 1980, and the Basanti Devi Amirchand Prize by the Indian Council of Medical Research (ICMR) in 1966. The 1976 World Health Assembly President, Sir Harold Walter, gave him the Leon Bernard Foundation Award [2].

## Review

Dr. Ramalingaswami's life and career

In the Indian state of Andhra Pradesh, he was born on August 8, 1921, His father was a civil servant whose name was V. Gumpaswami and his mother was V. Sundaramma. Many from his family worked as teachers, and the man he was named after, his paternal grandfather, was the headmaster of the most esteemed grammar school in the area and held an English literature degree from Madras University. It was at Andhra Medical College in Visakhapatnam that Dr. Ramalingaswami studied medicine. He completed his MBBS in 1944 and his MD in 1946. Following that, he was given a scholarship to Oxford University, where he graduated with a DPhil in 1951 and a DSc in 1967 [2].

After returning to India in 1947, he worked at the Nutrition Research Laboratory (NRL) in Coonoor, Nilgiris, which is now the National Institute of Nutrition, Hyderabad, for the rest of his life studying the causes and mechanisms of the diseases common in developing nations. His studies included nutritional anemia, iodine deficiency disorders, protein-energy malnutrition, and tropical liver diseases [4,5]. He worked as a professor of pathology and the director of the All India Institute of Medical Sciences (AIIMS) in 1969. During his 10 years there, he put much effort into creating a top-notch pathology school that drew in many bright students [3]. His knowledge of nutritional deficiencies was helpful during the great famine in Bihar in 1967 and the war in Bangladesh in 1970-1971, when millions of refugees needed food and rehabilitation.

Dr. Ramalingaswami’s works

His scientific writing was significant and a lot of his papers are still considered classics in their fields. He researched the prevalence of goiter in the Kangra Valley, a region home to over 100,000 people, while employed at the NRL. He found that the disease was drastically reduced by mixing potassium iodate with common salt [6]. The National Iodine Deficiency Control Programme was established as a result of his work, significantly improving public health.

The Kangra Valley study (1956-1972)

This was the pioneer study conducted in the Kangra District of Himachal Pradesh, India. Soon after joining the AIIMS, Dr. Ramalingaswami and his colleagues started a large-scale community-based study that involved over 100,000 people over the course of 16 years (1956-1972). These investigations demonstrated that iodine deficiency was the cause of endemic goiter. These results prompted the now-famous ‘Kangra Valley experiment’, demonstrating that endemic goiter could be prevented with a minimal dose of iodine after years of observation [6].

AIDS

Despite strong opposition from a variety of sources, Dr. Ramalingaswami emphasized that AIDS could spread like wildfire in the current social milieu prevalent in India and many developing countries. He organized testing facilities across India and attended conferences concerning this illness. He was a board member of the International AIDS Vaccine Initiative and supported the creation of both AIDS testing kits and a suitable AIDS vaccine. His work led to the establishment of collaboration in the necessary research efforts between the National AIDS Research Institute, the ICMR, and the International AIDS Vaccine Initiative [7].

Nutritional anemia

Along with his student Dr. S. K. Sood, Dr. Ramalingaswami started a comprehensive epidemiological study. It was discovered that the primary cause of anemia in pregnant women was iron deficiency. In keeping with his usual protocol, concurrent experimental investigations were carried out to elucidate the pathogenetic mechanism at play. The Indian government developed a National Nutritional Anemia Control Program based on these observations. The group conducted research outside of India in partnership with colleagues in Sri Lanka and South Africa; the findings were widely published in two WHO Technical Reviews series and International Nutritional Anemia Consultative Group publications [8].

Liver disease

Another significant contribution of his work was the research on the connection between primary hepatocellular carcinoma and the chronic hepatitis B virus. He expanded on research on toxic liver injury and Indian childhood cirrhosis, defined the entity of ‘non-cirrhotic portal fibrosis’, and debunked the notion that nutritional cirrhosis is caused by a virus [9].

Plague outbreak of 1994

In 1994, there were two separate plague outbreaks in India: one in Maharashtra, which was suspected to be the bubonic plague, and another in Surat, which is in the neighboring state of Gujarat, which was alleged to be the pneumonic plague. By October 1994, when the two outbreaks mainly had abated, the Indian government formed a Technical Advisory Committee on Plague, with Dr. Ramalingaswami serving as its chairman. This committee was established to confirm that the plague caused the events and strategize future actions that, using suitable epidemiological monitoring and supportive laboratory services, would decrease susceptibility to newly emerging and re-emerging infectious diseases.

The relationship between pneumonic and bubonic plague in the two outbreaks, the role of antibiotics in managing them, and the possibility of antibiotic resistance were all covered in detail by the Technical Advisory Committee in their extensive discussion of ways to strengthen the general framework for controlling infectious diseases. Dr. Ramalingaswami's organizational and intellectual leadership, as well as his concerns regarding the possible resurgence of infectious diseases and the emergence of brand-new ones, were evident in the committee's report [10-12].

Research contributions from the AIIMS era

Ramalingaswami's most important contributions were made in his little over 20 years of employment at the AIIMS, where he first served as a professor and department head (1957-1969) and then as the institute's director and professor of pathology (1969-1979). He fervently supported the idea that understanding the pathophysiology and pathology of common diseases in society required conducting excellent basic research. Thus, rather than being restricted to describing the disorder's pathology, his research was driven by a holistic approach to solving the associated issues [1,3].

Vitamin A deficiency and eye disease

At Oxford University, Ramalingaswami started researching vitamin A deficiency and carried on this research after returning to India. He gave an example of how vitamin A should be given to kids to prevent vision issues. He promoted national and international programs for controlling blindness [13].

Children's malnutrition in India

Despite having a higher national per capita income, higher levels of education, and more access to clean water, India is known for having a higher percentage of malnourished children than the sub-Saharan region. Dr. Ramalingaswami co-authored "The Asian Enigma," a perspective piece on this, with Jonsson and Rohde in 1996. It highlights the fact that women's deficient status compared to men was the cause of this predicament. It is commonly known that in the South Asian region, boys are typically given preference regarding food. As a result, an extremely high proportion of women in South Asia experience anemia and iron deficiency. Dr. Ramalingaswami rose to prominence as one of the most important advocates for women's rights [14].

International commitments

Over more than 50 years, Ramalingaswami's work was distinguished by a fundamental synthesis, one that is difficult to achieve. He undertook laboratory, clinical, and community-based research to address some of the most basic health issues facing his native India. These were pertinent to the entire developing world. International agencies working on these issues sought him out more than any other scientist. To investigate the dietary status of the Thai people, the WHO Regional Office for Southeast Asia dispatched Dr. Ramalingaswami to Thailand in 1955. He made a report, a fundamental document for comprehending the nutritional conditions that prevailed after traveling throughout Thailand. In December 1952, he attended his first international conference on iodine and thyroid deficiencies. He was then invited to participate in all international meetings related to this field, both scientific and policy-making. His research demonstrated that iodized salt could be added to food to prevent iodine deficiency disorders. This finding had an impact on international policymaking as well as Indian government policy [1,3].

Dr. Ramalingaswami worked on committees and policy-making bodies, addressing a wide range of nutrition-related issues, directing research and encouraging others to conduct the necessary research. His contributions were especially significant in understanding the pathophysiology of protein-energy malnutrition, a common condition that impairs young children's development. Dr. Ramalingaswami's contributions to understanding nutritional anemia made him famous anywhere the topic of mothers' health came up, whether it was at the United Nations Children's Fund (UNICEF) or the WHO. He consistently promoted the need for both the mother's and the child's health to be improved and for the mother to be given more authority to better care for her children. UNICEF appointed him as a Special Adviser to the Executive Director on Child Survival and Development (1988-89). The WHO invited him to their nutrition-related meetings, including those on diseases related to micronutrient deficiencies or malnutrition based on protein-energy imbalance. In the late 1950s, the WHO offered him a job as a nutrition advisor, which he declined. It was only in the 1990s that he accepted the position of Advisor to the Director-General of WHO, as organizing secretary for the International Conference on Nutrition held in Rome, Italy, in 1992, which was unique in its concept and organization [1,2].

Dr. Ramalingaswami went above and beyond the WHO's Alma Ata declaration, which strongly emphasized primary healthcare. He served as the chairman of the Joint Committee of the Indian Council of Social Science Research and the ICMR which resulted in the 1980 publication of ‘Health for All: An Alternate Strategy,' a report with significant implications for the health of the Indian populace. The health aspect of India's subsequent five-year plans was based on this report. Later, upon its formation, he joined the International Commission on Health Research for Development. He made innumerable trips around the globe to advocate for and highlight the health needs of underprivileged groups, women, children, and developing countries. He became close friends and personal acquaintances with numerous eminent scientists around the globe [2,3].

## Conclusions

Dr. Vulimiri Ramalingaswami was a multifaceted individual recognized for his work as a physician, educator, humanist, administrator, and research scientist. Throughout his research career, which spanned six decades, he advised the Indian government and numerous international organizations, including Harvard University, UNICEF, and the WHO. He was indeed a 20th-century legend.
